# Octopus-Inspired Biomimetic Annular Sealing Grooves: Design and Performance Optimization Under Extreme Conditions

**DOI:** 10.3390/biomimetics10050322

**Published:** 2025-05-16

**Authors:** Zhipeng Pan, Shijun Xu, Xiang Guan, Zhihong Wang, Zhenghai Qi, Xiangrui Ye, Jianyang Dong, Yongming Yao, Zhengzhi Mu

**Affiliations:** 1 School of Mechanical and Aerospace Engineering, Jilin University, Changchun 130022, China; panzp23@mails.jlu.edu.cn (Z.P.); guanxiang22@mails.jlu.edu.cn (X.G.); zhihong23@mails.jlu.edu.cn (Z.W.); qizh24@mails.jlu.edu.cn (Z.Q.); yexr24@mails.jlu.edu.cn (X.Y.); jydong24@mails.jlu.edu.cn (J.D.); 2 Shanghai Aerospace Equipment Manufacturer Co., Ltd., Shanghai 200245, China; crogeics_disk@163.com; 3 Key Laboratory of Bionic Engineering, Ministry of Education, Jilin University, Changchun 130022, China

**Keywords:** biomimetic sealing, octopus sucker, finite element analysis, annular sealing groove, hierarchical sealing structure

## Abstract

This study introduces an innovative annular sealing groove design inspired by the hierarchical structure of octopus suckers, addressing the limitations of conventional seals under extreme conditions in aerospace engineering. Using finite element analysis, eight bionic configurations with varying groove parameters (width, depth, number) were systematically evaluated under cryogenic (−196.25 °C) and high-pressure (2 MPa) scenarios. Results show that the optimized bionic6 configuration (seven grooves, 0.4 mm width, 0.4 mm depth) achieved a 21.71% improvement in average von Mises stress compared to the original design, demonstrating enhanced leakage resistance. Parameter interaction analysis revealed groove number as the most significant factor affecting performance, followed by width, while depth showed minimal influence. The hierarchical groove architecture effectively mimicked the multi-level sealing mechanism of octopus suckers, reducing leakage paths and improving adaptability to irregular surfaces. This work bridges biological inspiration and engineering application, providing a scalable solution for extreme environments. The identified optimal parameters lay a theoretical foundation for designing high-performance seals in aerospace, cryogenic storage, and advanced manufacturing.

## 1. Introduction

The development of high-performance sealing systems capable of maintaining performance under extreme conditions (e.g., cryogenic temperatures, high pressures, and irregular surfaces) remains a critical challenge in the aerospace, automotive, and energy industries [1,2,3]. Conventional sealing technologies, such as O-rings and lip seals, often fail under these conditions due to material embrittlement, reduced elasticity, or inadequate surface conformability [4,5,6]. Inspired by biological systems, researchers have increasingly turned to biomimicry to address these limitations [7,8]. Among the most complex adhesive systems in nature, octopus suction cups are capable of producing strong, reversible adhesion to a variety of surfaces [9]. The main reason for their ability to produce strong adhesion is the ability of their suction cups to form effective seals. At the same time, this seal can also accommodate topological irregularities, making it a promising template [10,11]. Octopus suckers are multifunctional organs consisting of a hierarchical structure of muscles, connective tissues, and sensory elements. The infundibulum, a disc-like structure with radial ridges and grooves, creates a seal by conforming to the surface topography [12].

Traditional sealing solutions rely on mechanical compression or chemical bonding, which are inherently limited by material properties and operational conditions. For instance, elastomeric seals often fail at cryogenic temperatures due to reduced ductility [4]. Additionally, their inability to dynamically adapt to surface irregularities leads to premature wear and leakage. Vacuum-based systems, while effective on smooth surfaces, require continuous energy input and struggle with irregular geometries. These limitations necessitate the development of adaptive, energy-efficient sealing solutions. In recent years, biomimetic sealing technologies have gained significant attention due to their potential to overcome the limitations of conventional sealing methods [13]. Researchers have drawn inspiration from various biological systems to develop innovative sealing solutions that can adapt to extreme conditions and complex surface geometries [14,15].

Hu [16] et al. designed a bionic sealing ring with non-smooth surface technology, which significantly improved the static and dynamic seal performance compared to traditional rectangular rings. Similarly, Zhang [17] et al. developed an octopus-inspired bionic flexible gripper for apple grasping, which exhibited strong sealing performance and adaptability to surface irregularities. The gripper’s design was based on the muscle structure and movement characteristics of octopus tentacles, allowing it to conform to complex surface geometries. The application of computational fluid dynamics (CFD) simulations has enabled researchers to optimize the performance of biomimetic sealing systems [18]. Han [19] et al. conducted a comprehensive study on the sealing performance of bionic sealing rings under various operating conditions. Their research demonstrated that the von Mises stress and contact stress distribution in bionic sealing rings could be optimized through structural design, leading to improved durability and reliability. Additionally, the study highlighted the importance of precompression, medium pressure, and friction coefficient in determining the sealing performance. Yan [20] et al. conducted a numerical simulation study on the sealing characteristics of different types of sealing rings used in centrifugal pumps. The study compared the leakage and energy consumption of straight seam, labyrinth, comb tooth, stepped, and rhombus sealing rings. The findings indicated that labyrinth, stepped, and comb tooth sealing rings exhibited better sealing performance with significantly lower leakage quantities. Another significant contribution to the field comes from Xi et al., who designed a bionic vacuum sucker inspired by the abdominal foot of abalone [21]. The sucker’s design incorporated sealing ring structures that enhanced adsorption force and stability. Experimental results showed that the bionic sucker with two sealing rings, a 1.5 mm sealing ring width, and a 3 mm sealing ring spacing exhibited a 15.8% higher adsorption force compared to standard suckers. The exploration of biomimetic surface textures has also yielded valuable insights. Chen [22] et al. investigated the effect of snake-biomimetic surface textures on finger sealing performance under hydrodynamic lubrication. Their numerical model, based on the Reynolds equation, revealed that texture orientation, depth, and density significantly influenced the hydrodynamic pressure and friction coefficient. The study concluded that longitudinal textures with shallow depths (<25 μm) and moderate density (20–40%) provided optimal sealing and anti-wear performance. Wang [23] et al. designed a bionic soft octopus sucker that could absorb soft tissues effectively. This design incorporated a stiffness gradient and acetabular protuberance, inspired by the structure of octopus suckers, resulting in improved adsorption force and adaptability to curved surfaces. The study demonstrated that the bionic sucker could increase adsorption force by 25.1% on cylindrical surfaces and 45.2% on spherical surfaces compared to traditional designs. The development of biomimetic sealing systems has also been driven by advances in materials science and manufacturing techniques. While existing bionic seal designs show potential [24,25,26], they have yet to fully replicate the hierarchical sealing efficiency observed in octopus suckers [27]. For example, previous studies have focused on biomimicry of the macrostructure of octopus suckers, leaving a gap in the sealing performance of surface morphology treatments under extreme conditions [28,29,30].

This study introduces an innovative annular sealing groove design inspired by the microstructural hierarchy of octopus suckers. By integrating multiple concentric grooves with optimized dimensions, the proposed structure aims to enhance leakage resistance and adaptability to irregular surfaces. Through finite element analysis (FEA), we systematically evaluate the sealing performance of eight bionic configurations under cryogenic and high-pressure conditions, identifying the optimal parameters for practical applications. Our work bridges the gap between biological inspiration and engineering implementation, offering a scalable solution for extreme environments.

## 2. Structural Characterization and Adsorption Mechanism of Octopus Suckers

### 2.1. Biological Structure of Octopus Suckers

**Figure 1:**
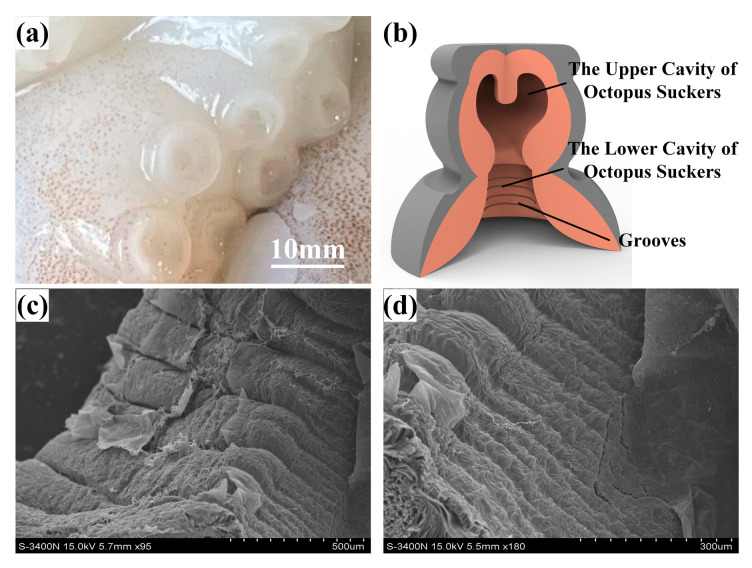
Octopus suction cup structure and surface characteristics. (**a**) Structure of the octopus suction cup. (**b**) Suction cup model. (**c**,**d**) Surface characteristics of octopus cups.

From a macroscopic perspective, the octopus sucker exhibits a disk-like structure, comprising a central cavity, encircling ring muscles, and an outer elastic folded epidermis, as illustrated in Figure 1a. Figure 1b illustrates the structural composition of the suction cup. It comprises an upper chamber and a lower chamber, with the surface of the lower chamber featuring a grooved structure. At the microscopic level, the disk of the octopus sucker exhibits a non-uniform surface texture, characterized by the presence of subtle groove structures, as illustrated in Figure 1c,d.

Negative pressure generated in the central cavity directly influences octopus tentacle adhesion strength, while surface furrows facilitate hierarchical sealing to maintain this pressure gradient. Initially, the outermost furrows engage with the substrate surface to primarily fill macroscopic asperities, thereby minimizing leakage paths. As adhesion strength increases, inner furrows progressively conform to the surface topography, establishing a hierarchical sealing architecture. Through this mechanism, gas or liquid attempting to leak through the suction cup–substrate interface must traverse multiple microchannels partitioned by furrows. Each furrow-generated microcompartment acts as a sealing barrier, substantially increasing the complexity and length of leakage paths while significantly reducing leak probability. This hierarchical sealing architecture is considered one of the key factors enabling octopus suction cups to maintain high-efficiency adhesion and sealing performance under complex environmental conditions. Furthermore, it provides a valuable biological prototype for bionic sealing technologies, inspiring researchers to develop advanced sealing materials or devices by mimicking the microscopic furrow structures of octopus suckers to meet industrial requirements for high sealing performance. Surface furrow depth and width of ten selected octopus suckers were measured under identical field-of-view conditions using a fully automated stereomicroscope self-measurement system, as presented in Table 1.

### 2.2. Adsorption Mechanism

When the octopus applies its sucker to a substrate, circumferential muscle contraction rapidly expands the central cavity volume, reducing internal pressure to generate negative pressure. According to fluid mechanics principles, the higher external pressure relative to internal cavity pressure generates robust adhesive force that secures the sucker to the substrate. Surface furrows on the sucker form a multi-level sealing architecture that minimizes fluid leakage, maintaining stable negative pressure within the cavity.

## 3. Innovative Design of Annular Sealing Grooves Inspired by Octopus Suckers

### 3.1. Methodology Transfer

**Figure 2:**
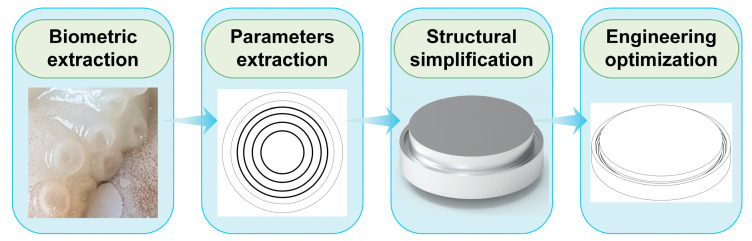
Transfer framework.

As shown in Figure 2, biometric feature extraction, parametric abstraction, structural simplification, and engineering optimization are used as the transfer framework. The core features of the octopus sucker (hierarchical groove structure, multi-stage sealing mechanism) are mapped to with the sealing groove design. The microstructure of the grooves on the octopus suction cup surface is measured to characterize their features, and bionic principles derived from these features are applied to the sealing structure to optimize groove design for actual workpieces.

### 3.2. Sealing Structure

**Figure 3:**
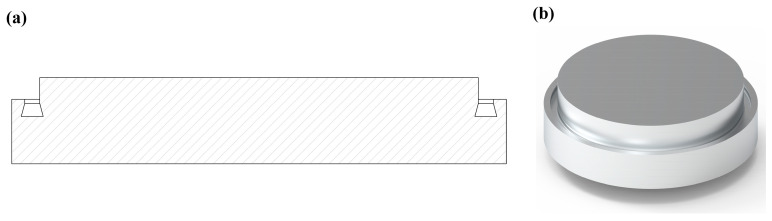
Seal valve plate. (**a**) Sealed valve section. (**b**) Sealed valve model drawing.

The simplified model is shown in Figure 3a,b. The seals have grooves designed to make contact with the trifluoroethylene to form a sealing structure; these sealing grooves have a narrow dovetailed top and bottom cross-section.

### 3.3. Structural Design Innovations

Inspired by octopus sucker microstructures, multiple concentric annular grooves are incorporated to replicate the hierarchical sealing architecture observed in biological systems. This configuration increases leakage path complexity and length through multi-level contact interfaces, enhancing sealing reliability. As detailed in Table 2, mature octopus sucker furrows exhibit width and depth ranging from 0.1 mm to 0.5 mm. Direct application of these dimensions to precision miniature sealing components excessively roughens the surface, compromising structural integrity and complicating analysis of non-smooth groove effects on seal performance. To address this, a biomimetic scaling strategy was implemented based on pneumatic solenoid valve specifications: non-smooth features were retained while reducing the groove widths or depths to 0.2 mm and 0.4 mm.

Due to the difference in surface groove morphology and deformation ability between the sealing groove and those of an octopus suction cup, there is a gap. Therefore, the bionic pressure glue groove layout can’t be completely in accordance with the groove degree of density of the octopus suction cup. Changing the number of grooves in the circumferential direction on the bottom surface of the suction cup alters the sparseness of its non-smooth surface morphology. This is also conducive to ANSYS finite element analysis and the processing of the suction cup mold. Therefore, the number of grooves of a specific shape on the inner surface of the suction cup was selected as a reference factor for evaluating the degree of non-smoothness. Based on the common characteristics of mature octopus suction cups, which typically feature multiple distinct grooves, the groove design aimed to meet the principle of reasonable spacing (not too sparse or too dense). Two models were designed with four and seven circular grooves, respectively, with spacings of 1.4 mm and 0.7mm. In each case, the grooves are evenly distributed along the inner surface of the rubber groove. This consideration is based on the actual size of the sealing structure, and it aims to minimize influence on the original strength.

Based on standard sucker dimensional parameters and two selected biomimetic design features, the bionic annular sealing groove was developed by integrating optimized geometric dimensions (Table 2) derived from octopus-inspired structural analysis. When selecting the size parameters, the main consideration is to preferably minimize the impact on the original seal strength. Although a smaller groove has a lesser impact on strength, it also increases machining difficulty. Therefore, the parameters in the table are chosen after comprehensive consideration.

**Figure 4:**
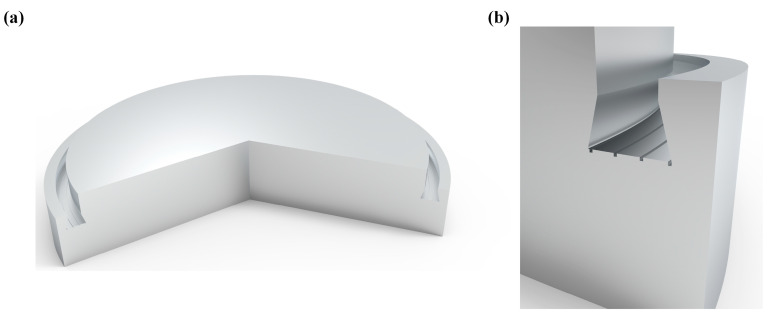
Bionic seal valve plate. (**a**) Bionic seal valve plate structure schematic. (**b**) Bionic seal valve plate cross-section.

The three-dimensional model of the bionic annular sealing groove established according to the characteristic parameters in Table 2 is shown in Figure 4.

### 3.4. Innovation in Sealing Principle

Multiple sealing mechanism simulation: The octopus suction cup realizes multiple seals through the wrinkled skin. The annular sealing groove draws on this principle and sets up multiple sealing grooves to form a multiple sealing structure. In the pipeline connection seal, multiple annular sealing grooves cooperate with the sealing ring to effectively prevent the leakage of liquid or gas and can maintain good sealing performance even under severe working conditions such as high pressure and low temperature.

### 3.5. Prototype Implementation

The sealing ring is fabricated from polychlorotrifluoroethylene (PCTFE), whose low-temperature toughness and chemical stability satisfy the demands of extreme environments. The sealing structure is constructed from aluminum alloy, with the entire assembly manufactured by pressing high-temperature molten PCTFE powder onto the structure and cooling it to form a monolithic component.

## 4. Performance Research Based on Simulation Tests

### 4.1. Simulation Analysis Method

In order to select the bionic annular sealing groove with excellent sealing performance, the finite element simulation software ANSYS2024R1 was used to analyze the force on the bottom surface of the sealing rubber ring during sealing. The sealing rubber ring is circular, with a diameter of 109 mm and a thickness of 10 mm. The surface has a sealing groove combined with the bottom surface of the sealing pressure groove. The material is selected as polychlorotrifluoroethylene, with a density of 2130 kg/cm^3^, a thermal expansion coefficient of 6 × 10^−5^ k^−1^, a Young’s modulus of 1.7 × 10^3^ Mpa, and a Poisson’s ratio of 0.3. The material of the sealing matrix is aluminum alloy, and the aluminum alloy material is selected in the material library. Since the sealing of the sealing groove is a contact problem, the bottom surface of the sealing rubber ring is set as the target surface, and the bottom surface of the sealing pressure groove is set as the contact surface. In the analysis, the sealing substrate was set to a fixed constraint, and then 2 Mpa pressure was applied to the surface of the sealing rubber ring while the temperature was set to −196.25 degrees Celsius. The bionic structure is consistent with the parameter settings of the standard sealing structure.

**Figure 5:**
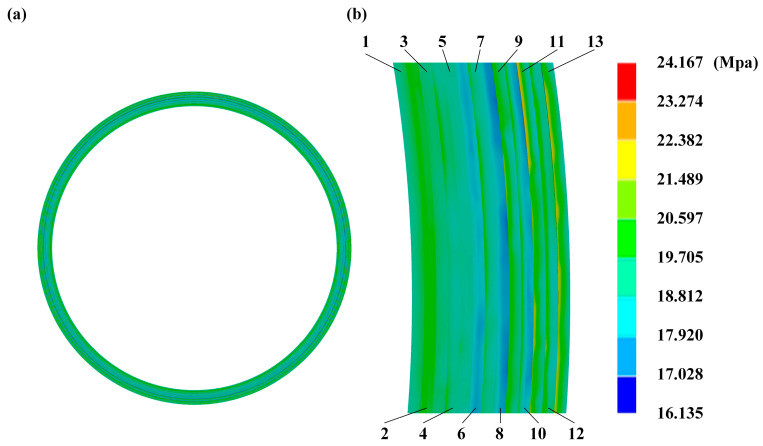
Stress distribution on the underside of the flap. (**a**) Stress distribution on the lower surface. (**b**) Schematic division of the area.

The von Mises stress cloud diagram of the bottom surface of the sealing ring can be obtained from the simulation results, and the von Mises stress of 13 regions is measured from the center to the edge along the radial direction of the sealing ring using the probe function, as shown in Figure 5. The selected 13 regions are consistent on different sealing structures. The von Mises stress values of the 13 regions of the bottom surface of the bionic and standard sealing rubber rings are shown in Table 3.

### 4.2. Simulation Results and Analysis

**Figure 6:**
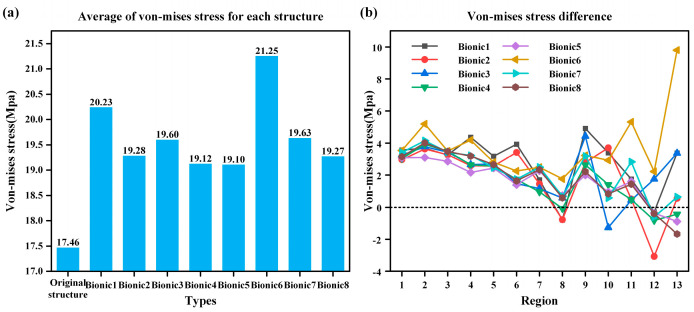
Stress conditions on the underside of the flap. (**a**) Von Mises stress averages. (**b**) Von Mises stress distribution by region.

Through the simulation test, the pressure distribution of the bionic annular sealing groove and the original annular sealing groove under the same working conditions is obtained. Figure 6a shows the average value of the von Mises stresses of the original annular seal groove and each bionic annular seal groove. As observed in Figure 6a, the average von Mises stress of the bionic structures was higher than that of the original annular seal groove, indicating enhanced sealing performance through bionic structure. Among them, bionic6 has the highest effect improvement, and the sealing effect is increased by 21.71%. Figure 6b illustrates the differences in von Mises stress between the bionic annular seal grooves and the original structure in different regions. Notably, the bionic configurations in regions 1–7 and 9 exhibited higher von Mises stress compared to the original design, while moderate improvements were observed in other areas. This further validates the enhanced sealing performance achieved through bionic structure.

**Figure 7:**
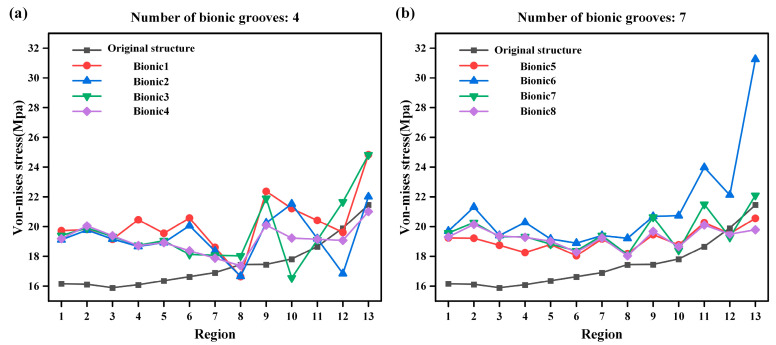
Comparison of different bionic seal groove number von Mises stress cases. (**a**) The number of bionic sealing grooves is 4 von Mises stress case. (**b**) The number of bionic sealing grooves is 7 von Mises stress case.

Figure 7a,b each show the von Mises stress distribution of each region with four and seven bionic grooves, respectively. It can be noted that when the number of bionic grooves is four, the effect of multiple bionic models in regions 8, 10, 12, and 13 is lower than that of the original model, while when the number is seven, the effect is significantly improved, and only the partial bionic models in regions 12 and 13 are lower than the original model. When comparing Figure 7a,b, it can be noted that the structure with seven bionic grooves exhibits better von Mises stress performance and overall stability than that of the structure with four bionic grooves.

**Figure 8:**
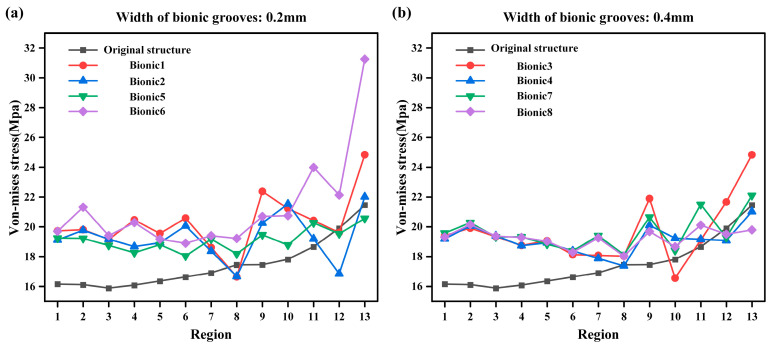
Comparison of different bionic seal groove width von Mises stress cases. (**a**) The width of bionic sealing grooves is 0.2mm von Mises stress case. (**b**) The width of bionic sealing grooves is 0.4mm von Mises stress case.

Figure 8a,b each show the equivalent stress distribution in each region with a bionic groove width of 0.2 mm and 0.4 mm, respectively. It can be noted that when the bionic groove width is 0.2 mm, the effect of multiple bionic models in regions 8, 12, and 13 is lower than that of the original model. Similarly, when the width is 0.4mm, the effect of partial bionic models in regions 10, 12, and 13 is lower than that of the original model. When comparing Figure 8a,b, it can be noted that in the region where the performance is lower than that of the original model, the maximum difference in von Mises stress between the structure with a bionic groove width of 0.4 mm and the original structure is smaller than the difference observed with a bionic groove width of 0.2 mm. At the same time, it can be noted that the stability of both bionic groove widths is improved in regions 1–7, but deteriorates in regions 8–13. Overall, the stability of the structure with a width of 0.4 mm is better than that of the structure with a width of 0.2 mm.

**Figure 9:**
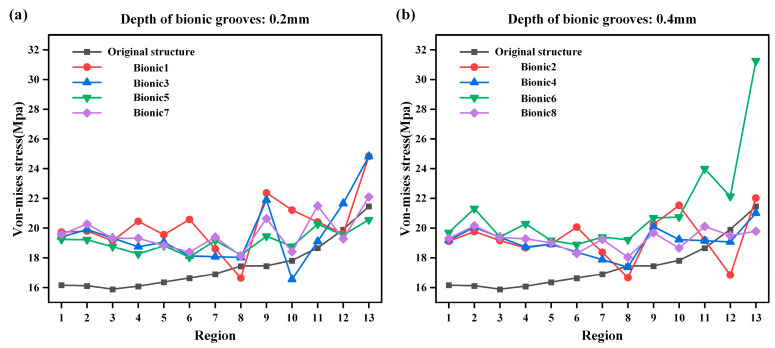
Comparison of different bionic seal groove depth von Mises stress cases. (**a**) The depth of bionic sealing grooves is 0.2mm von Mises stress case. (**b**) The depth of bionic sealing grooves is 0.4mm von Mises stress case.

Figure 9a,b each show the equivalent stress distribution in each region with a bionic groove height of 0.2 mm and 0.4 mm, respectively. It can be noted that when the height of the bionic groove is 0.2 mm, the effect of multiple bionic models in regions 8, 10, 12, and 13 is lower than that of the original model. Similarly, when the width is 0.4mm, the effect of the bionic model in regions 8, 12, and 13 is lower than that of the original model. Comparing Figure 9a,b, it can be noted that both structures with modified bionic groove heights show areas where the von Mises stress is lower than that of the original structure, but the overall effect is improved to a certain extent. At the same time, it can be noted that the stability of the two bionic groove heights is similar. The above description shows that bionic groove heights of 0.2 mm and 0.4 mm result in similar improvements in sealing performance.

**Figure 10:**
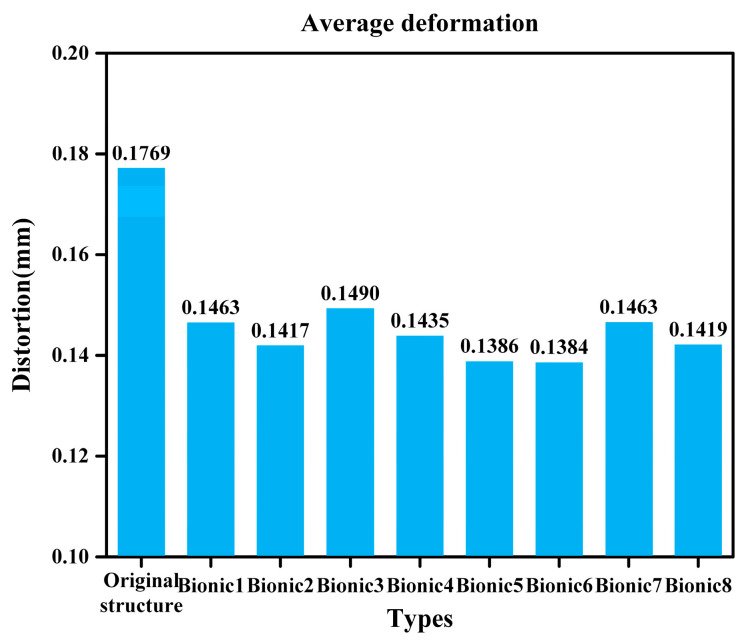
Average deformation.

Moreover, we have also calculated the average deformation of the contact surface. It was found that with the incorporation of the bionic structure, the average deformation has generally decreased. And the smaller the deformation is, the more advantageous it is for the performance of the sealing structure, as shown in Figure 10.

In summary, optimized bionic grooves enhance sealing performance in most areas, especially when configured with seven grooves and a width of 0.4 mm. In terms of parameter interactions, groove quantity offers the greatest effect on overall performance, followed by width, whereas height contributes negligibly. For applications requiring high parameter stability, the 0.4-mm width combined with the seven-groove structure is preferred to balance local and system-wide performance.

## 5. Conclusions

This study introduces an innovative annular sealing groove design inspired by octopus sucker microstructures to address the limitations of conventional seals under extreme conditions. Through finite element analysis (FEA), eight bionic configurations with varying groove parameters (width, depth, number) were systematically evaluated under cryogenic (−196.25 °C) and high-pressure (2 MPa) scenarios. The optimized bionic6 configuration (seven grooves, 0.4 mm width, 0.4 mm depth) demonstrated a 21.71% improvement in average von Mises stress compared to the original design, highlighting superior leakage resistance. Notably, groove number exerted the most significant influence on performance, followed by width, while depth showed negligible effects; the seven-groove structure with 0.4 mm width achieved the best balance between local stress distribution and overall stability. The hierarchical groove architecture effectively replicated the multi-level sealing mechanism of octopus suckers, minimizing leakage paths and enhancing conformability to irregular surfaces. This work bridges biological inspiration and engineering implementation, offering a scalable solution for extreme environments. The identified optimal parameters provide a theoretical foundation for designing high-performance seals in aerospace, cryogenic storage, and advanced manufacturing, with future research opportunities focusing on experimental validation, dynamic load testing, and integration with smart materials.

## Figures and Tables

**Table 1:** Octopus Suction Cup Recess Size Chart.

Suction Cup Serial Number	Recess Depth H (mm)	Notch Width L (mm)
1	0.19	0.16
2	0.23	0.17
3	0.39	0.38
4	0.25	0.19
5	0.17	0.21
6	0.40	0.16
7	0.28	0.30
8	0.35	0.31
9	0.30	0.22
10	0.42	0.33

Note: The octopus used to measure the dimensions of the suction cup grooves in this study is the common octopus. This species is widely distributed in the tropical and temperate waters of the world’s oceans, including many regions of the Atlantic, Pacific, and Indian Oceans. Its sucker structure is representative of similar species and is frequently employed in research on biological adhesion and sealing mechanisms. The common octopus inhabits offshore areas with rocky reefs, caves, and muddy substrates, which offer an abundant food source and a suitable living environment. Due to its extensive distribution and ease of access, as well as the uniqueness and typicality of its sucker structure, it serves as an ideal subject for studying biological adhesion and sealing mechanisms. Such research can assist scientists in gaining a more profound understanding of the special functions of organisms in nature and provide inspiration for the development of related engineering fields.

**Table 2:** Bionic seal groove size table.

Serial Number	Groove Width L (mm)	Groove Depth H (mm)	Number of Grooves	Groove Spacing (mm)
bionic1	0.2	0.2	4	1.4
bionic2	0.2	0.4	4	1.4
bionic3	0.4	0.2	4	1.4
bionic4	0.4	0.4	4	1.4
bionic5	0.2	0.2	7	0.7
bionic6	0.2	0.4	7	0.7
bionic7	0.4	0.2	7	0.7
bionic8	0.4	0.4	7	0.7

**Table 3:** Von Mises stress values in different regions of the sealing structure (Mpa).

Region	1	2	3	4	5	6	7	8	9	10	11	12	13
initial model	21.457	19.910	18.672	17.825	17.465	17.452	16.914	16.646	16.370	16.096	15.895	16.128	16.157
bionic1	19.728	19.807	19.152	20.462	19.551	20.580	18.613	16.646	22.376	21.219	20.418	19.617	24.840
bionic2	19.119	19.765	19.169	18.678	18.933	20.063	18.366	16.669	20.254	21.532	19.190	16.848	22.012
bionic3	19.407	19.904	19.337	18.750	19.058	18.125	18.076	18.027	21.895	16.556	19.111	21.663	24.829
bionic4	19.194	20.051	19.392	18.733	18.929	18.381	17.876	17.371	20.098	19.232	19.156	19.080	21.015
bionic5	19.241	19.224	18.755	18.260	18.803	18.048	19.178	18.183	19.464	18.780	20.269	19.533	20.56
bionic6	19.700	21.315	19.404	20.287	19.172	18.897	19.399	19.217	20.694	20.746	23.987	22.13	31.255
bionic7	19.574	20.275	19.321	19.323	18.828	18.383	19.403	18.127	20.645	18.409	21.498	19.283	22.100
bionic8	19.310	20.147	19.372	19.281	19.022	18.289	19.251	18.034	19.679	18.664	20.108	19.499	19.789

## Data Availability

The original contributions presented in this study are included in the article. Further inquiries can be directed to the corresponding author.
